# Data regarding fracture incidence according to fracture site, month, and age group obtained from the large public health insurance claim database in Japan

**DOI:** 10.1016/j.dib.2019.103780

**Published:** 2019-03-06

**Authors:** Shuichiro Hayashi, Tatsuya Noda, Shinichiro Kubo, Tomoya Myojin, Yuichi Nishioka, Tsuneyuki Higashino, Manabu Akahane, Tomoaki Imamura

**Affiliations:** a Department of Public Heath, Health Management and Policy, Nara Medical University, 840 Shijo-cho Kashihara-shi, Nara, 634-8521, Japan; b Management Innovation Division, Consulting Unit, Mitsubishi Research Institute, Inc., 2-10-3 Nagata-cho, Chiyoda-ku, Tokyo, 100-8141, Japan

**Keywords:** Epidemiology, Fracture, Season, National database, Health insurance claims, Japan

## Abstract

The National Database of Health Insurance Claims and Specific Health Checkups of Japan includes all health insurance claims submitted in Japan and is considered representative of almost all health claims in Japan. Data regarding fracture incidence, based on the documented diagnoses in the claims and relevant procedure codes, were extracted from the National Database of Health Insurance Claims and Specific Health Checkups of Japan. This data paper includes fracture incidence according to fracture site, month, and age group for the population in Kanto area (Tokyo and surrounding areas), which consists of approximately 42 million people. These data provide supplementary material to be interpreted for the article “Variation in Fracture Risk by Season and Weather: A Comprehensive Analysis across Age and Fracture Site Using a National Database of Health Insurance Claims in Japan” Hayashi et al., and serve as one of the largest epidemiological datasets regarding seasonal differences in fracture incidence according to fracture site and age group.

**Table:** Specifications table^1^

Subject area	*Orthopedic surgery, Healthcare-related database*
More specific subject area	*Epidemiology of fracture*
Type of data	*Table*
How data was acquired	*Extracted from national database of health insurance claims*
Data format	*Analyzed*
Experimental factors	*Health insurance claims by providers were collected and accumulated in the government database between April 2013 and March 2016*
Experimental features	*Data extracted from the government database were analyzed*
Data source location	*Kanto Area (Tokyo and surrounding areas), Japan*
Data accessibility	*Data are in this article*
Related research article	Hayashi S, Noda T, Kubo S, Myojin M, Nishioka Y, Higashino T, Imamura T. Variation in Fracture Risk by Season and Weather: A Comprehensive Analysis across Age and Fracture Site Using a National Database of Health Insurance Claims in Japan. Bone 120 (2019) 512–518. https://doi.org/10.1016/j.bone.2018.12.014[1].

**Table:** 

**Value of the Data** • The dataset consisted of comprehensive epidemiological data of fractures across all age groups and fracture sites. • The incidence of fractures was described in a large population of >40 million, based on one of the world's largest health databases. • This is one of the largest datasets describing the seasonal variation of fracture incidence, including >500,000 fracture cases. • The codes and algorithms used to extract data from the database are described in this article for greater transparency and reproducibility. • These data could be used as a benchmark in epidemiological research into fractures, because of the scale and completeness of the sample.

## 1 Data

The data described in this article represent the number of cases of peripheral fractures stratified according to fracture site, calendar month, and age group, based on health insurance claims submitted by the healthcare providers for the population of approximately 42 million in Kanto area (Tokyo and surrounding areas) in Japan between April 2013 and March 2016 (Table 1, Table 2). The dataset provides comprehensive coverage on the incidence of peripheral fractures, and contains the incidences of all peripheral fracture sites and all age groups, from children (0–19 years) to the elderly (≥80 years). The data also describe the incidences of fractures for each calendar month, providing quantitative data for seasonal variation of fracture incidences. Cases involving fractures were extracted from the National Database of Health Insurance Claims and Specific Health Checkups of Japan (NDB), one of the largest healthcare-related databases in the world. The total number of fracture cases in the data was 508,051. The cases for this data were extracted from the NDB using diagnosis codes and procedure codes specific to fractures. The codes and algorithms used to extract data from the NDB are shown for transparency and reproducibility of the data [2]. The data contains all health insurance claims submitted in the area and is representative of the incidence of the population.

**Table 1:** Area populations and numbers of cases.

	Age group (years)	Population^a^ (in thousands)	Number of fracture cases
Men			
	0–19	3,700	79,756
	20–39	5,513	28,985
	40–64	7,522	45,394
	65–79	3,563	30,560
	≥80	970	26,580
	Total	21,268	211,275
Women			
	0–19	3,512	34,019
	20–39	5,137	11,329
	40–64	7,195	46,180
	65–79	3,961	84,423
	≥80	1,714	120,825
	Total	21,519	296,776
Total			
	0–19	7,212	113,775
	20–39	10,650	40,314
	40–64	14,717	91,574
	65–79	7,524	114,983
	≥80	2,684	147,405
	Total	42,787	508,051

a Population is based on publicly available data from the Ministry of Internal Affairs and Communication, Japan.

**Table 2:** Number of fracture cases observed between April 17, 2013 and March 15, 2016.

Age groups	Fracture site	Number of observed cases											
		Calendar month											
		Jan	Feb	Mar	Apr	May	Jun	Jul	Aug	Sep	Oct	Nov	Dec
0–19 years	Rib or sternum	≤10	15	13	≤10	13	16	16	13	18	16	13	16
	Clavicle, scapula, or humerus	1283	1278	1278	1510	2297	2024	1685	1367	1885	1984	1685	1389
	Radius or ulna—distal radius	1678	1629	1990	2224	3653	2912	2513	1743	2749	2892	2327	1830
	Radius or ulna—other	899	932	985	1203	1932	1769	1401	978	1547	1610	1258	1016
	Hand bone	1907	2148	2000	1818	2548	2777	2217	1473	2467	2791	2630	2309
	Femur—hip	≤10	≤10	≤10	≤10	≤10	≤10	≤10	≤10	13	12	≤10	≤10
	Femur—other	53	49	64	48	69	66	59	51	67	58	62	56
	Patella, tibia, or fibula	687	601	650	600	765	640	557	441	618	665	683	708
	Ankle	491	480	450	436	475	465	397	319	405	461	466	460
	Foot bone—toe	297	309	281	286	464	513	562	475	478	438	398	370
	Foot bone—other	372	398	338	339	462	413	382	317	456	450	457	373
20–39 years	Rib or sternum	68	62	47	51	49	60	60	52	50	53	56	53
	Clavicle, scapula, or humerus	613	660	578	383	506	399	474	502	455	471	417	445
	Radius or ulna—distal radius	561	708	469	240	332	294	268	302	271	282	235	359
	Radius or ulna—other	273	339	282	203	264	256	223	221	239	251	210	227
	Hand bone	839	808	787	750	1010	1023	984	914	919	992	940	914
	Femur—hip	59	33	34	24	34	31	38	37	48	38	34	33
	Femur—other	38	45	40	40	47	41	46	38	53	40	45	41
	Patella, tibia, or fibula	414	408	327	319	427	358	377	363	336	362	362	364
	Ankle	316	367	273	280	395	317	310	302	316	320	318	348
	Foot bone—toe	194	190	173	157	252	252	299	282	297	273	259	260
	Foot bone—other	305	272	280	282	408	389	395	404	370	354	347	292
40–64 years	Rib or sternum	271	236	199	178	259	211	213	238	214	243	234	264
	Clavicle, scapula, or humerus	1243	1205	986	886	1284	1107	1074	1145	1116	1165	1098	1204
	Radius or ulna—distal radius	2050	2060	1173	1016	1335	1298	1398	1385	1367	1386	1449	1731
	Radius or ulna—other	575	580	382	373	485	434	495	399	451	490	477	543
	Hand bone	1177	1084	969	856	1136	1104	1100	1081	1136	1224	1146	1181
	Femur—hip	563	491	431	366	436	401	414	439	468	468	485	503
	Femur—other	82	90	85	60	74	79	74	84	84	116	79	89
	Patella, tibia, or fibula	1202	1224	932	744	853	744	798	831	871	914	976	1116
	Ankle	868	927	642	519	701	644	597	616	658	634	667	756
	Foot bone—toe	492	431	399	400	559	585	740	775	734	704	613	548
	Foot bone—other	653	599	583	579	731	724	833	855	803	810	729	761
65–79 years	Rib or sternum	248	220	213	186	190	177	173	218	207	212	227	297
	Clavicle, scapula, or humerus	1355	1219	991	882	1011	1048	1111	1065	1083	1225	1248	1351
	Radius or ulna—distal radius	3366	3412	2001	1905	2479	2360	2508	2403	2468	2770	2713	3064
	Radius or ulna—other	682	695	481	514	623	588	598	582	604	713	663	710
	Hand bone	698	707	544	544	664	586	630	602	656	766	736	857
	Femur—hip	2756	2454	2002	1692	2019	1857	1848	1920	2114	2309	2377	2687
	Femur—other	215	192	150	122	183	191	166	184	162	189	193	216
	Patella, tibia, or fibula	1136	1118	840	812	867	767	812	769	886	990	1052	1147
	Ankle	594	674	448	411	454	425	436	463	445	515	529	546
	Foot bone—toe	203	164	139	144	232	242	312	334	316	278	236	233
	Foot bone—other	486	467	406	425	558	515	608	548	538	603	577	604
≥80 years	Rib or sternum	215	180	145	150	174	157	145	142	159	200	228	231
	Clavicle, scapula, or humerus	1310	1193	903	849	1012	1017	1067	998	1147	1272	1282	1393
	Radius or ulna—distal radius	2250	2051	1502	1410	1757	1594	1742	1713	1811	2037	2025	2191
	Radius or ulna—other	491	443	394	359	421	413	453	449	468	533	497	494
	Hand bone	447	369	324	284	329	308	311	346	349	447	437	514
	Femur—hip	8711	7579	6452	5973	6932	6562	6817	6808	6829	7919	7964	8785
	Femur—other	371	365	293	278	316	321	333	331	365	368	368	429
	Patella, tibia, or fibula	557	514	448	393	444	411	410	414	448	516	490	603
	Ankle	182	191	115	129	150	134	138	140	128	171	167	184
	Foot bone—toe	62	49	41	53	61	72	99	82	hi94	95	56	58
	Foot bone—other	196	162	146	146	178	174	189	208	189	197	240	206
Number of days during the study period		93	85	77	74	93	90	93	93	90	93	90	93

## 2 Experimental design, materials, and methods

### 2.1 Extraction of data from the original database

NDB is a database of all monthly claims of public health insurance in Japan, including all procedural codes, International Statistical Classification of Diseases, Tenth Edition (ICD-10) codes, and prescriptions, across inpatient and outpatient services. Because of the wide coverage of public health insurance, the NDB is considered representative of almost all health claims in Japan. We applied to use the NDB as members of a research group funded by Health Science and Labor Research Grant from the Ministry of Health, Labour and Welfare, Japan, and permission was granted. We also obtained approval by the appropriate Institutional Review Board. An isolated database was created in the research group and consisted of claim data collected from the original NDB database between April 2013 and March 2016.

### 2.2 Matching more than one claim to the same individual

Although the NDB used two personal identification variables (hereafter referred to as ID1 and ID2) to link individual patients’ insurance claims, the efficiency of this process was limited. Therefore, we used another identification variable (hereafter referred to as ID0), which was created by applying a patient-matching algorithm based on the ID1 and ID2 variables, as described previously [3].

### 2.3 Inclusion criteria for the claim data

Claims that fulfilled the inclusion criteria (Table 3) were extracted from our database with the ID0, procedural code, date of application for the procedure, date of hospitalization (if applicable), ICD-10 code, date of documentation for ICD-10 code, prefecture code, and age-group code. Fracture sites were then classified according to the fracture sites listed in Table 2B, using the ICD-10 codes in the claims.

**Table 3:** Criteria for the extraction of claims from the original database.

Purpose	Criteria for extraction (Claims that fulfilled all criteria were extracted)
Dataset A Data for extracting cases	1. Claims for both inpatient and outpatient services, submitted by clinics or hospitals located in Kanto area (Tokyo and the six surrounding prefectures) 2. Claims that included the treatment procedure codes listed in Table 2A 3. Claims that included one of the ICD-10 codes listed in Table 2B as a principal or secondary diagnosis but not as a suspected diagnosis 4. Claims covering April 1, 2013 to March 31, 2016
Dataset B Data for refining the date of the first visit to health care providers	1. Claims submitted by clinics or hospitals located in Kanto area (Tokyo and the six surrounding prefectures) 2. Claims that included one of the ICD-10 codes listed in Table 2B as a principal or secondary diagnosis but not as a suspected diagnosis 3. Claims that were not included in Dataset A 4. Claims covering April 1, 2013 to March 31, 2016

ICD-10 = International Classification of Diseases, Tenth Edition.

### 2.4 Definition of cases

A case was defined as the first incidence of fracture to one of the sites shown in Table 2B between April 15, 2013, and March 17, 2016. Fracture incidence included records of claims with the fracture-specific treatment codes shown in Table 4A and the ICD-10 codes shown in Table 4B. Cases involving multiple fractures were considered single cases if multiple fractures occurred in only one group of sites; fractures that occurred in different groups of sites were classed separately for each group. Recurrent fracture cases that occurred in the same group of sites were excluded.

**Table 4A:** List of all procedural codes for data extraction.

Procedural Codes a	Name of procedures
K044	Closed reduction of fracture
K045	Percutaneous pinning of fracture
K046	Open reduction and internal fixation of fracture
K046-2	Open reduction and internal fixation of periprosthetic fracture
K073	Open reduction of intra-articular fracture
K073-2	Arthroscopic reduction of intra-articular fracture
K078	Arthrodesis
K081	Hemiarthroplasty
K082	Total arthroplasty

a Procedural codes are indicated based on the claim system for health insurance in Japan. The list of procedural codes was provided by the Ministry of Health, Labour and Welfare, Japan. (https://www.mhlw.go.jp/file/06-Seisakujouhou-12400000-Hokenkyoku/0000114822.pdf)

**Table 4B:** List of ICD-10 codes for data extraction and the grouping of fracture sites.

Fracture site	ICD-10 codes
Clavicle, scapula, or humerus	S42, S49.7
Radius or ulna	
Distal radius	S52.5
Other	S52.1–4/6–9, S59.7
Hand bone	S62, S69.7
Femur	
Hip	S72.0–2
Other	S72.3–9, S79.7
Patella, tibia, or fibula	S82.0–4/7/9, S89.7
Ankle	S82.5–6/8
Foot bone	
Toe	S92.4–5
Other	S92.0–3/6–9, S99.7

^1^ Abbreviations: ICD-10: International Statistical Classification of Diseases, Tenth Edition; NDB: National Database of Health Insurance Claims and Specific Health Checkups of Japan.

### 2.5 Exclusion criteria

Cases in which any pair of the following days, the day of documentation of diagnosis, the day of application of the treatment procedure, or the day of hospitalization, were more than two weeks apart were excluded in an attempt to omit hospital-acquired cases of fractures but include nosocomial fracture cases in which the documentation of diagnosis or treatment occurred several days after admission.

### 2.6 Definition of the date of fracture incidence

The date of the first visit to a hospital/clinic for a fracture was considered a proxy for the date of fracture, as the claim data did not include the date of injury.

We created two interim datasets, Dataset A and Dataset B, to accurately describe the date of first visit for the fractures for clinics/hospitals. Dataset A was created for collecting an accurate number of cases by including cases if both medical procedures and clinical diagnoses met the criteria. Dataset B was created for refining the date of the first visit to health care providers. Dataset B included claims of the patients who visited other facilities for fractures, regardless of the medical procedures conducted. The claims included in Dataset A were limited to those containing specific medical procedure codes, but patients might be referred from other facilities where a diagnosis had been made a few days prior. We aimed to describe the date of the first visit for clinics/hospitals for the fractures by matching claims of the same individual between Dataset A and Dataset B without compromising the specificity of the diagnosis.

The earliest date on which the documentation of diagnosis, the application of the treatment procedure, or hospitalization occurred according to Dataset A was defined as the date of the first visit, as long as there was no claim involving the documentation of fracture diagnosis in the same group of sites in another hospital/clinic in Dataset B within the previous 14 days. For cases in which claims included the documentation of fractures in the same group of sites in other hospitals/clinics in Dataset B within the previous 14 days, the date of the earlier visit was considered the date of the first visit.

### 2.7 Statistical analysis

The numbers of fracture cases were accumulated and stratified according to the group of fracture sites involved, based on ICD-10 classification, and sub-classified into the following five age groups: 0**–**19, 20–39, 40–64, 65–79, and ≥80 years. All analyses were performed using SPSS (version 24). The terms of use for the NDB prevented us from reporting fracture sites with ≤10 cases, to protect patient privacy. For fracture sites with lower incidence rates, the upper limits of the incidence ranges were reported instead of precise values.
